# 1-(5-Amino-2,4-dinitro­phen­yl)pyridinium chloride monohydrate

**DOI:** 10.1107/S1600536812038834

**Published:** 2012-09-15

**Authors:** Rajamanickam Babykala, Doraisamyraja Kalaivani

**Affiliations:** a PG and Research Department of Chemistry, Seethalakshmi Ramaswami College, Tiruchirappalli 620 002, Tamil Nadu, India

## Abstract

In the cation of the title hydrated salt, C_11_H_9_N_4_O_4_^+^·Cl^−^·H_2_O, the six-membered rings are inclined to each other at 79.0 (1)° and an intra­molecular N—H⋯O hydrogen bond occurs. In the crystal, N—H⋯Cl hydrogen bonds link two cations and two anions into centrosymmetric group, and O—H⋯Cl hydrogen bonds involving the water mol­ecules further link these groups into chains in [101]. An O—H⋯O inter­action is also present. The water mol­ecule is disordered over two sets of sites in a 0.555 (13):0.445 (13) ratio

## Related literature

For applications of *N*-substituted pyridinium salts, see: Sliwa (1996 ▶); Ali *et al.* (2005 ▶); Chelossi *et al.* (2006 ▶); Azzouz *et al.* (2008 ▶). For related structures, see: Shmidt *et al.* (2005 ▶); Wojtas *et al.* (2006 ▶); Manickkam & Kalaivani (2011 ▶); Chernyshev *et al.* (2011 ▶); Sridevi & Kalaivani (2012 ▶).
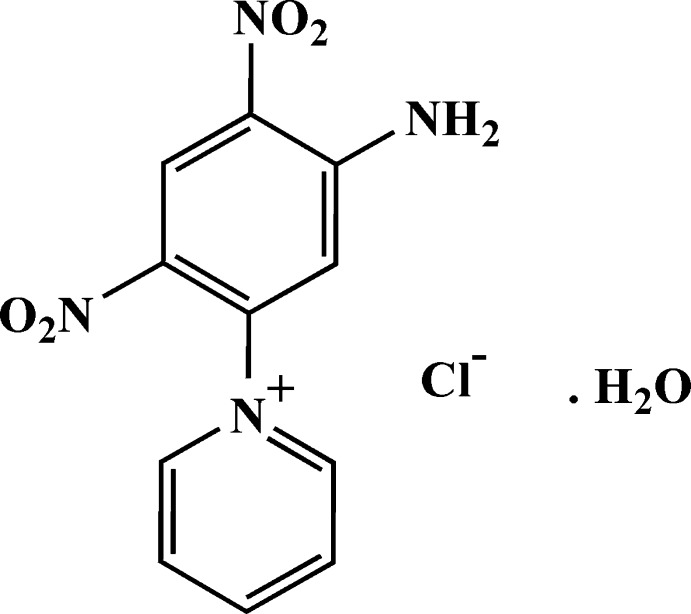


## Experimental

### 

#### Crystal data

C_11_H_9_N_4_O_4_^+^·Cl^−^·H_2_O
*M* *_r_* = 314.69
Monoclinic, 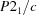

*a* = 5.4312 (4) Å
*b* = 21.493 (2) Å
*c* = 11.3892 (9) Å
β = 92.362 (3)°
*V* = 1328.33 (19) Å^3^
*Z* = 4
Mo *K*α radiation
μ = 0.32 mm^−1^
*T* = 293 K
0.30 × 0.25 × 0.20 mm

#### Data collection

Bruker Kappa APEXII CCD diffractometer
Absorption correction: multi-scan (*SADABS*; Bruker, 2004 ▶) *T*_min_ = 0.871, *T*_max_ = 0.939
14754 measured reflections
3125 independent reflections
2288 reflections with *I* > 2σ(*I*)
*R*_int_ = 0.031

#### Refinement

*R*[*F* ^2^ > 2σ(*F* ^2^)] = 0.038
*wR*(*F* ^2^) = 0.112
*S* = 1.03
3125 reflections
224 parameters
9 restraints
H atoms treated by a mixture of independent and constrained refinement
Δρ_max_ = 0.20 e Å^−3^
Δρ_min_ = −0.27 e Å^−3^

### 

Data collection: *APEX2* (Bruker, 2004 ▶); cell refinement: *APEX2* and *SAINT* (Bruker, 2004 ▶); data reduction: *SAINT* and *XPREP* (Bruker, 2004 ▶); program(s) used to solve structure: *SIR92* (Altomare *et al.*, 1993 ▶); program(s) used to refine structure: *SHELXL97* (Sheldrick, 2008 ▶); molecular graphics: *ORTEP-3* (Farrugia, 1997 ▶) and *Mercury* (Macrae *et al.*, 2008 ▶); software used to prepare material for publication: *PLATON* (Spek, 2009 ▶).

## Supplementary Material

Crystal structure: contains datablock(s) global, I. DOI: 10.1107/S1600536812038834/cv5319sup1.cif

Structure factors: contains datablock(s) I. DOI: 10.1107/S1600536812038834/cv5319Isup2.hkl

Supplementary material file. DOI: 10.1107/S1600536812038834/cv5319Isup3.cml

Additional supplementary materials:  crystallographic information; 3D view; checkCIF report

## Figures and Tables

**Table 1:** Hydrogen-bond geometry (Å, °)

*D*—H⋯*A*	*D*—H	H⋯*A*	*D*⋯*A*	*D*—H⋯*A*
N4—H4*A*⋯O4	0.86 (2)	2.09 (2)	2.671 (2)	124 (2)
N4—H4*B*⋯Cl1	0.87 (2)	2.35 (2)	3.2162 (19)	171 (2)
N4—H4*A*⋯Cl1^i^	0.86 (2)	2.56 (2)	3.2268 (16)	135 (2)
O5—H5*B*⋯Cl1	0.90 (2)	2.34 (3)	3.187 (3)	158 (5)
O5—H5*A*⋯Cl1^ii^	0.93 (2)	2.51 (2)	3.429 (9)	169 (5)
O5′—H5*D*⋯O5^iii^	0.91 (2)	1.94 (3)	2.815 (16)	160 (5)

Symmetry codes: (i) 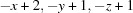; (ii) 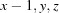; (iii) 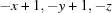.

**Fig. 1.**
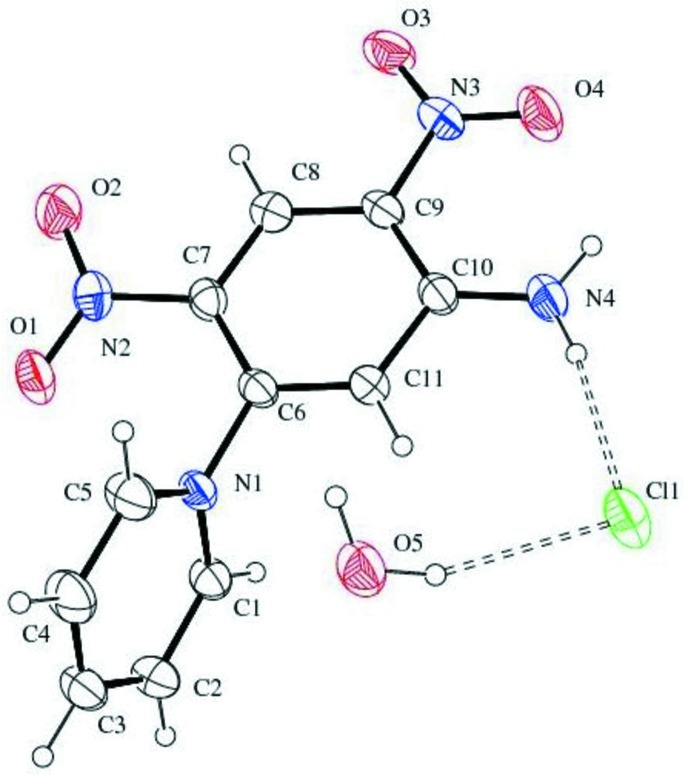
The content of asymmetric unit of the title compound showing the atomic labelling and 40% probability displacement ellipsoids. Dashed lines denote hydrogen bonds. Only major component of the disordered water molecule is shown.

**Fig. 2.**
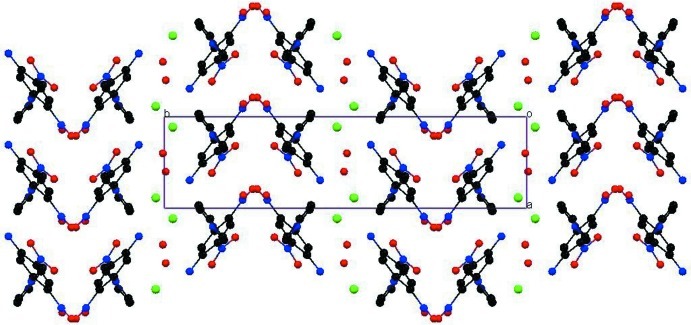
A portion of the crystal packing viewed down the c axis and showing the herring bone arrangement of the molecules. Only major components of the disordered water molecules are shown. H atoms were omitted for clarity.

## supplementary crystallographic information

### Comment

N-Substitued pyridinium salts are widely used in organic synthesis (Azzouz *et al.*, 2008), medicinal field (Chelossi *et al.*, 2006), electrodeposition (Ali *et al.*, 2005) and dye preparations (Sliwa, 1996). As a continuation of our studies of new substituted pyridinium barbiturates (Manickkam & Kalaivani, 2011; Sridevi & Kalaivani, 2012), we report the crystal structure of the title compound, (I).

In (I) (Fig. 1), all bond lengths and angles are normal and correspond to those observed in the related compounds (Shmidt *et al.*, 2005; Wojtas *et al.*, 2006; Chernyshev *et al.*, 2011). In the cation, the pyridine ring is twisted notably from the dinitrophenyl ring and the dihedral angle between their planes is 78.93 (5)°. The two nitro groups, O1—N2—O2 and O3—N3—O4, deviate from the benzene ring at 7.97 (10)° and 4.18 (17)°, respectively. Intermolecular N—H···Cl and O—H···Cl hydrogen bonds (Table 1) consolidate the crystal packing. The overall molecular packing forming a herring bone arrangement when view down *c* axis is shown in Fig. 2.

### Experimental

Analytical grade 1,3-dichloro-4,6-dinitrobenzene (DCDNB) and barbituric acid were used as supplied by Aldrich company. Pyridine was distilled under reduced pressure and the fraction boiling over at its boiling point was used for the preparation of the title molecular salt.DCDNB (2.01 g, 0.01 mol) in 15 ml absolute ethanol was mixed with barbituric acid (1.28 g, 0.01 mol) in 30 ml of absolute ethanol. Pyridine (3.16 g, 0.04 mol)was added to the above mixture which was heated to 40°C and shaken well for 5–6 hrs. The solution was kept as such at room temperature for 48 hrs. On standing dark violet colour crystals separate out.After filtering out these violet crystals, the filterate was kept as such at room temperature (25°C).From the filtrate, one of the by-products of the reaction between DCDNB, barbituric acid and pyridine, separates as pale greenish yellow crystals after 3 months. These crystals were powdered well, and washed with copious amount of ethanol and dry ether, recrystallized from absolute alcohol and subjected to single-crystal X-ray analysis. Yield: 40–50%; m.p.: 508 K.

### Refinement

C-bound H atoms were positioned geometrically (C—H 0.93 Å), and refined as riding, with U_iso_(H) = 1.2 U_eq_(C). N- and O-bound H atoms were located on a difference map, and refined with restraints N—H = 0.88 (2) Å, O—H = 0.92 (2) Å.

### Crystal data

**Table:**

C_11_H_9_N_4_O_4_^+^·Cl^−^·H_2_O	*F*(000) = 648
*M**_r_* = 314.69	*D*_x_ = 1.574 Mg m^−^^3^
Monoclinic, *P*2_1_/*c*	Mo *K*α radiation, λ = 0.71073 Å
Hall symbol: -P 2ybc	Cell parameters from 4581 reflections
*a* = 5.4312 (4) Å	θ = 2.6–25.4°
*b* = 21.493 (2) Å	µ = 0.32 mm^−^^1^
*c* = 11.3892 (9) Å	*T* = 293 K
β = 92.362 (3)°	Block, red
*V* = 1328.33 (19) Å^3^	0.30 × 0.25 × 0.20 mm
*Z* = 4	

### Data collection

**Table:**

Bruker Kappa APEXII CCD diffractometer	3125 independent reflections
Radiation source: fine-focus sealed tube	2288 reflections with *I* > 2σ(*I*)
Graphite monochromator	*R*_int_ = 0.031
ω and φ scan	θ_max_ = 27.9°, θ_min_ = 2.6°
Absorption correction: multi-scan (*SADABS*; Bruker, 2004)	*h* = −7→6
*T*_min_ = 0.871, *T*_max_ = 0.939	*k* = −28→28
14754 measured reflections	*l* = −11→14

### Refinement

**Table:**

Refinement on *F*^2^	Primary atom site location: structure-invariant direct methods
Least-squares matrix: full	Secondary atom site location: difference Fourier map
*R*[*F*^2^ > 2σ(*F*^2^)] = 0.038	Hydrogen site location: inferred from neighbouring sites
*wR*(*F*^2^) = 0.112	H atoms treated by a mixture of independent and constrained refinement
*S* = 1.03	*w* = 1/[σ^2^(*F*_o_^2^) + (0.0538*P*)^2^ + 0.2604*P*] where *P* = (*F*_o_^2^ + 2*F*_c_^2^)/3
3125 reflections	(Δ/σ)_max_ = 0.001
224 parameters	Δρ_max_ = 0.20 e Å^−^^3^
9 restraints	Δρ_min_ = −0.27 e Å^−^^3^

### Special details

**Table:**

Geometry. All e.s.d.'s (except the e.s.d. in the dihedral angle between two l.s. planes) are estimated using the full covariance matrix. The cell e.s.d.'s are taken into account individually in the estimation of e.s.d.'s in distances, angles and torsion angles; correlations between e.s.d.'s in cell parameters are only used when they are defined by crystal symmetry. An approximate (isotropic) treatment of cell e.s.d.'s is used for estimating e.s.d.'s involving l.s. planes.
Refinement. Refinement of *F*^2^ against ALL reflections. The weighted *R*-factor *wR* and goodness of fit *S* are based on *F*^2^, conventional *R*-factors *R* are based on *F*, with *F* set to zero for negative *F*^2^. The threshold expression of *F*^2^ > σ(*F*^2^) is used only for calculating *R*-factors(gt) *etc*. and is not relevant to the choice of reflections for refinement. *R*-factors based on *F*^2^ are statistically about twice as large as those based on *F*, and *R*- factors based on ALL data will be even larger.

### Fractional atomic coordinates and isotropic or equivalent isotropic displacement parameters (Å^2^)

**Table:**

	*x*	*y*	*z*	*U*_iso_*/*U*_eq_	Occ. (<1)
C1	0.3483 (3)	0.66419 (8)	0.10183 (14)	0.0449 (4)	
H1	0.4780	0.6878	0.1336	0.054*	
C2	0.3105 (3)	0.66065 (9)	−0.01760 (15)	0.0531 (4)	
H2	0.4149	0.6814	−0.0671	0.064*	
C3	0.1174 (4)	0.62626 (9)	−0.06342 (15)	0.0563 (5)	
H3	0.0871	0.6242	−0.1443	0.068*	
C4	−0.0302 (4)	0.59508 (11)	0.01082 (17)	0.0663 (6)	
H4	−0.1607	0.5712	−0.0194	0.080*	
C5	0.0137 (3)	0.59893 (10)	0.12967 (16)	0.0613 (5)	
H5	−0.0855	0.5773	0.1803	0.074*	
C6	0.2469 (3)	0.63860 (8)	0.29921 (13)	0.0403 (4)	
C7	0.1136 (3)	0.67842 (8)	0.37070 (14)	0.0414 (4)	
C8	0.1804 (3)	0.68200 (8)	0.48832 (14)	0.0441 (4)	
H8	0.0915	0.7076	0.5370	0.053*	
C9	0.3760 (3)	0.64842 (8)	0.53520 (13)	0.0432 (4)	
C10	0.5080 (3)	0.60594 (8)	0.46686 (13)	0.0439 (4)	
C11	0.4326 (3)	0.60318 (8)	0.34576 (14)	0.0446 (4)	
H11	0.5138	0.5761	0.2969	0.054*	
N2	−0.0913 (3)	0.71602 (7)	0.32853 (13)	0.0493 (4)	
N1	0.1997 (2)	0.63388 (6)	0.17294 (11)	0.0404 (3)	
N3	0.4403 (3)	0.65911 (7)	0.65862 (12)	0.0517 (4)	
O1	−0.1404 (2)	0.71820 (7)	0.22235 (12)	0.0628 (4)	
O2	−0.2092 (3)	0.74376 (8)	0.40064 (13)	0.0787 (5)	
O3	0.3182 (3)	0.69609 (7)	0.71230 (11)	0.0676 (4)	
O4	0.6154 (3)	0.63139 (8)	0.70329 (11)	0.0740 (4)	
N4	0.6928 (3)	0.57003 (8)	0.50422 (14)	0.0560 (4)	
O5	0.3996 (12)	0.4960 (2)	0.1460 (6)	0.0653 (17)	0.555 (13)
O5'	0.4978 (12)	0.5075 (2)	0.0942 (7)	0.0622 (16)	0.445 (13)
Cl1	0.88637 (9)	0.47657 (2)	0.30769 (4)	0.06455 (18)	
H5A	0.274 (7)	0.487 (3)	0.196 (5)	0.15 (2)*	0.555 (13)
H5B	0.556 (4)	0.494 (4)	0.174 (5)	0.15 (2)*	0.555 (13)
H5C	0.520 (14)	0.481 (3)	0.156 (4)	0.08 (3)*	0.445 (13)
H5D	0.554 (9)	0.499 (3)	0.022 (2)	0.064 (16)*	0.445 (13)
H4A	0.747 (3)	0.5711 (10)	0.5766 (14)	0.063 (6)*	
H4B	0.759 (4)	0.5437 (9)	0.4563 (18)	0.071 (7)*	

### Atomic displacement parameters (Å^2^)

**Table:**

	*U*^11^	*U*^22^	*U*^33^	*U*^12^	*U*^13^	*U*^23^
C1	0.0445 (9)	0.0485 (9)	0.0413 (8)	−0.0051 (7)	−0.0018 (7)	0.0032 (7)
C2	0.0619 (11)	0.0584 (11)	0.0389 (9)	−0.0033 (8)	0.0029 (8)	0.0053 (8)
C3	0.0658 (12)	0.0667 (12)	0.0356 (8)	0.0059 (9)	−0.0056 (8)	0.0004 (8)
C4	0.0556 (11)	0.0906 (15)	0.0512 (11)	−0.0181 (10)	−0.0147 (9)	−0.0037 (10)
C5	0.0503 (10)	0.0871 (15)	0.0460 (9)	−0.0243 (10)	−0.0043 (8)	0.0058 (9)
C6	0.0384 (8)	0.0504 (9)	0.0318 (7)	−0.0074 (7)	−0.0034 (6)	0.0046 (6)
C7	0.0376 (8)	0.0440 (9)	0.0423 (8)	−0.0045 (6)	−0.0038 (6)	0.0045 (7)
C8	0.0464 (9)	0.0448 (9)	0.0412 (8)	−0.0039 (7)	0.0016 (7)	0.0002 (7)
C9	0.0481 (9)	0.0496 (9)	0.0315 (7)	−0.0067 (7)	−0.0022 (6)	0.0051 (7)
C10	0.0417 (8)	0.0514 (9)	0.0380 (8)	−0.0035 (7)	−0.0038 (7)	0.0073 (7)
C11	0.0418 (8)	0.0567 (10)	0.0353 (8)	0.0023 (7)	−0.0001 (6)	0.0029 (7)
N2	0.0442 (8)	0.0472 (8)	0.0556 (9)	−0.0020 (6)	−0.0096 (7)	0.0005 (7)
N1	0.0377 (6)	0.0501 (8)	0.0328 (6)	−0.0028 (6)	−0.0047 (5)	0.0041 (5)
N3	0.0631 (9)	0.0549 (9)	0.0365 (7)	−0.0051 (7)	−0.0052 (7)	0.0035 (7)
O1	0.0581 (8)	0.0727 (9)	0.0557 (8)	0.0073 (6)	−0.0222 (6)	−0.0006 (7)
O2	0.0757 (10)	0.0902 (11)	0.0696 (9)	0.0342 (8)	−0.0052 (8)	−0.0106 (8)
O3	0.0897 (10)	0.0747 (9)	0.0384 (7)	0.0102 (8)	0.0017 (7)	−0.0052 (6)
O4	0.0846 (10)	0.0864 (11)	0.0486 (8)	0.0167 (8)	−0.0252 (7)	−0.0053 (7)
N4	0.0577 (9)	0.0683 (11)	0.0409 (8)	0.0125 (8)	−0.0111 (7)	0.0022 (8)
O5	0.066 (3)	0.075 (2)	0.054 (3)	0.0075 (19)	−0.014 (2)	−0.0065 (17)
O5'	0.057 (3)	0.079 (2)	0.050 (3)	0.0092 (19)	0.001 (2)	0.002 (2)
Cl1	0.0775 (3)	0.0563 (3)	0.0576 (3)	0.0081 (2)	−0.0250 (2)	0.0010 (2)

### Geometric parameters (Å, º)

**Table:**

C1—N1	1.336 (2)	C8—H8	0.9300
C1—C2	1.369 (2)	C9—C10	1.414 (2)
C1—H1	0.9300	C9—N3	1.4528 (19)
C2—C3	1.369 (3)	C10—N4	1.323 (2)
C2—H2	0.9300	C10—C11	1.424 (2)
C3—C4	1.365 (3)	C11—H11	0.9300
C3—H3	0.9300	N2—O2	1.2176 (19)
C4—C5	1.367 (3)	N2—O1	1.2285 (18)
C4—H4	0.9300	N3—O4	1.2158 (19)
C5—N1	1.336 (2)	N3—O3	1.216 (2)
C5—H5	0.9300	N4—H4A	0.864 (15)
C6—C11	1.354 (2)	N4—H4B	0.874 (16)
C6—C7	1.403 (2)	O5—H5A	0.930 (19)
C6—N1	1.4540 (19)	O5—H5B	0.90 (2)
C7—C8	1.375 (2)	O5'—H5C	0.916 (19)
C7—N2	1.442 (2)	O5'—H5D	0.913 (19)
C8—C9	1.374 (2)		
			
N1—C1—C2	120.36 (15)	C8—C9—C10	121.78 (14)
N1—C1—H1	119.8	C8—C9—N3	116.37 (15)
C2—C1—H1	119.8	C10—C9—N3	121.85 (14)
C3—C2—C1	119.33 (16)	N4—C10—C9	126.50 (15)
C3—C2—H2	120.3	N4—C10—C11	118.14 (16)
C1—C2—H2	120.3	C9—C10—C11	115.35 (14)
C4—C3—C2	119.29 (16)	C6—C11—C10	122.40 (16)
C4—C3—H3	120.4	C6—C11—H11	118.8
C2—C3—H3	120.4	C10—C11—H11	118.8
C3—C4—C5	120.05 (18)	O2—N2—O1	123.14 (15)
C3—C4—H4	120.0	O2—N2—C7	118.02 (14)
C5—C4—H4	120.0	O1—N2—C7	118.83 (15)
N1—C5—C4	119.83 (17)	C1—N1—C5	121.11 (14)
N1—C5—H5	120.1	C1—N1—C6	118.62 (13)
C4—C5—H5	120.1	C5—N1—C6	120.26 (13)
C11—C6—C7	120.64 (14)	O4—N3—O3	122.92 (14)
C11—C6—N1	116.52 (14)	O4—N3—C9	118.69 (15)
C7—C6—N1	122.83 (14)	O3—N3—C9	118.39 (15)
C8—C7—C6	118.53 (14)	C10—N4—H4A	121.1 (14)
C8—C7—N2	117.52 (15)	C10—N4—H4B	120.2 (15)
C6—C7—N2	123.95 (14)	H4A—N4—H4B	119 (2)
C9—C8—C7	121.18 (15)	H5A—O5—H5B	119 (3)
C9—C8—H8	119.4	H5A—O5—H5C	119 (5)
C7—C8—H8	119.4	H5C—O5'—H5D	122 (4)
			
N1—C1—C2—C3	0.7 (3)	N4—C10—C11—C6	−179.34 (16)
C1—C2—C3—C4	−1.4 (3)	C9—C10—C11—C6	−0.2 (2)
C2—C3—C4—C5	0.7 (3)	C8—C7—N2—O2	−7.3 (2)
C3—C4—C5—N1	0.8 (3)	C6—C7—N2—O2	172.42 (16)
C11—C6—C7—C8	1.9 (2)	C8—C7—N2—O1	173.29 (15)
N1—C6—C7—C8	−176.69 (14)	C6—C7—N2—O1	−6.9 (2)
C11—C6—C7—N2	−177.90 (15)	C2—C1—N1—C5	0.9 (3)
N1—C6—C7—N2	3.5 (2)	C2—C1—N1—C6	179.68 (16)
C6—C7—C8—C9	1.2 (2)	C4—C5—N1—C1	−1.6 (3)
N2—C7—C8—C9	−178.98 (14)	C4—C5—N1—C6	179.61 (18)
C7—C8—C9—C10	−3.9 (2)	C11—C6—N1—C1	−78.17 (19)
C7—C8—C9—N3	175.86 (15)	C7—C6—N1—C1	100.44 (18)
C8—C9—C10—N4	−177.62 (17)	C11—C6—N1—C5	100.64 (19)
N3—C9—C10—N4	2.6 (3)	C7—C6—N1—C5	−80.8 (2)
C8—C9—C10—C11	3.3 (2)	C8—C9—N3—O4	−178.50 (16)
N3—C9—C10—C11	−176.45 (14)	C10—C9—N3—O4	1.3 (2)
C7—C6—C11—C10	−2.4 (2)	C8—C9—N3—O3	0.6 (2)
N1—C6—C11—C10	176.28 (14)	C10—C9—N3—O3	−179.63 (15)

### Hydrogen-bond geometry (Å, º)

**Table:**

*D*—H···*A*	*D*—H	H···*A*	*D*···*A*	*D*—H···*A*
N4—H4*A*···O4	0.86 (2)	2.09 (2)	2.671 (2)	124 (2)
N4—H4*B*···Cl1	0.87 (2)	2.35 (2)	3.2162 (19)	171 (2)
N4—H4*A*···Cl1^i^	0.86 (2)	2.56 (2)	3.2268 (16)	135 (2)
O5—H5*B*···Cl1	0.90 (2)	2.34 (3)	3.187 (3)	158 (5)
O5—H5*A*···Cl1^ii^	0.93 (2)	2.51 (2)	3.429 (9)	169 (5)
O5′—H5*D*···O5^iii^	0.91 (2)	1.94 (3)	2.815 (16)	160 (5)

Symmetry codes: (i) −*x*+2, −*y*+1, −*z*+1; (ii) *x*−1, *y*, *z*; (iii) −*x*+1, −*y*+1, −*z*.
